# Interferon‐γ and IL‐5 associated cell‐mediated immune responses to HPV16 E2 and E6 distinguish between persistent oral HPV16 infections and noninfected mucosa

**DOI:** 10.1002/cre2.396

**Published:** 2021-01-09

**Authors:** Anna Paaso, Hanna‐Mari Koskimaa, Marij J. P. Welters, Katja Kero, Jaana Rautava, Kari Syrjänen, Sjoerd H. van der Burg, Stina Syrjänen

**Affiliations:** ^1^ Department of Oral Pathology and Oral Radiology, Institute of Dentistry, Faculty of Medicine University of Turku Turku Finland; ^2^ Department of Clinical Oncology Leiden University Medical Center Leiden The Netherlands; ^3^ Department of Obstetrics and Gynecology Turku University Hospital Turku Finland; ^4^ Department of Oral and Maxillofacial Diseases, Clinicum University of Helsinki and Helsinki University Hospital Helsinki Finland; ^5^ Department of Clinical Research Biohit Oyj Helsinki Finland; ^6^ Department of Pathology Turku University Hospital Turku Finland

**Keywords:** cell‐mediated immunity, cytokines, human papillomavirus, oral cavity

## Abstract

**Objectives:**

Natural history of human papillomavirus (HPV) infection in the head and neck region is poorly understood, and their impact on collective HPV‐specific immunity is not known.

**Materials and methods:**

In this study, we have performed a systematic analysis of HPV16‐specific cell‐mediated immunity (CMI) in 21 women with known oral and genital HPV DNA status and HPV serology (Ab) based on 6‐year follow‐up data. These women being a subgroup from the Finnish Family HPV Study were recalled for blood sampling to be tested for their CMI‐responses to HPV16 E2, E6, and E7 peptides.

**Results:**

The results showed that HPV16 E2‐specific lymphocyte proliferation was more prevalent in women who tested HPV16 DNA negative in oral mucosa and were either HPV16 seropositive or negative than in HPV16 DNA+/Ab+ women (*p* = 0.046 and *p* = 0.035). In addition, the HPV16 DNA−/Ab− women most often displayed E6‐specific proliferation (*p* = 0.020). Proportional cytokine profiles indicated that oral HPV16‐negative women were characterized by prominent IFN‐γ and IL‐5 secretion not found in women with persisting oral HPV16 (*p* = 0.014 and *p* = 0.040, respectively).

**Conclusions:**

Our results indicate that the naturally arising immune response induced by oral HPV infections displays a mixed Th1/Th2/Th17 cytokine profile while women with persisting oral HPV16 might have an impaired HPV16‐specific CMI, shifted partly toward a Th2 profile, similarly as seen earlier among patients with high‐grade genital HPV lesions. Thus, the lack of HPV 16 E2 and E6 specific T memory cells and Th2 cytokines might also predispose women for persistent oral HPV16 infection which might be related to the risk of cancer.

## 1 INTRODUCTION

Human papillomavirus (HPV) infection with high‐risk genotypes is the main etiological factor of cervical cancer (CC), being present in over 90% of the cases. HPV can also infect head and neck region which includes oral cavity, oropharynx, nasopharynx, hypopharynx, and larynx. HPV is known to be associated with (i) totally benign lesions, (ii) potentially malignant lesions, and (iii) a subgroup of squamous cell carcinomas (HNSCC) (Rautava & Syrjänen, 2011; Syrjänen, 2003; Syrjänen et al., 2007). According to a recent meta‐analysis, approximately 22%, 50%, and 21% of oral, oropharyngeal, and laryngeal cancers are associated with HPV, mostly with the HPV16 genotype (Ndiaye et al., 2014).

Studies on the natural history of HPV in the head and neck region are scarce (Kero et al., 2012; Kero et al., 2014; Louvanto et al., 2010; Pierce Campbell et al., 2015; Rautava et al., 2012). Even less is known on the role of HPV‐specific immunity in oral and oropharyngeal HPV infections. In our previous study on the males in the Finnish Family HPV cohort, oral HPV infections were associated with HPV type‐specific humoral immune responses (Syrjänen et al., 2015), but this association was not found in women (Paaso et al., 2011).

The cell‐mediated immunity (CMI) caused by HPV infection is incompletely understood (de Jong et al., 2002; de Jong et al., 2004; Koskimaa et al., 2014; Paaso et al., 2015; van der Burg et al., 2007; Welters et al., 2003). We have studied HPV16‐specific CMI in women who developed cervical intraepithelial neoplasia (CIN) during a 10‐year follow‐up (FU), using constantly HPV‐negative women as a reference. While both the CIN and the genitally always negative control women exhibited HPV16‐specific CMI, the two groups could be distinguished on the basis of their IL‐17A secretion after HPV16 E6 stimulation (Paaso et al., 2015). There are hardly any studies on CMI immunity in the context of oral HPV infections. However, one study showed that local HPV‐specific T cells were more often present or less suppressed in HPV‐induced HNSCCs than in CCs (Heusinkveld et al., 2012).

The aim of the present study was to assess the association between oral HPV16 infection and immunity against HPV16. We analyzed women (the cases) with oral HPV16 infection who were HPV16L1 seropositive or ‐negative during the 6‐year FU. The control group consisted of women who remained constantly HPV16 DNA‐negative in their oral brush samples during the FU, being either HPV16‐seropositive or ‐negative. These four subgroups (DNA+/Ab+, DNA+/Ab−, DNA−/Ab+, DNA−/Ab−) were analyzed to disclose any differences in their HPV‐specific CMI.

## 2 METHODS

### 2.1 Women

**FIGURE 1:**
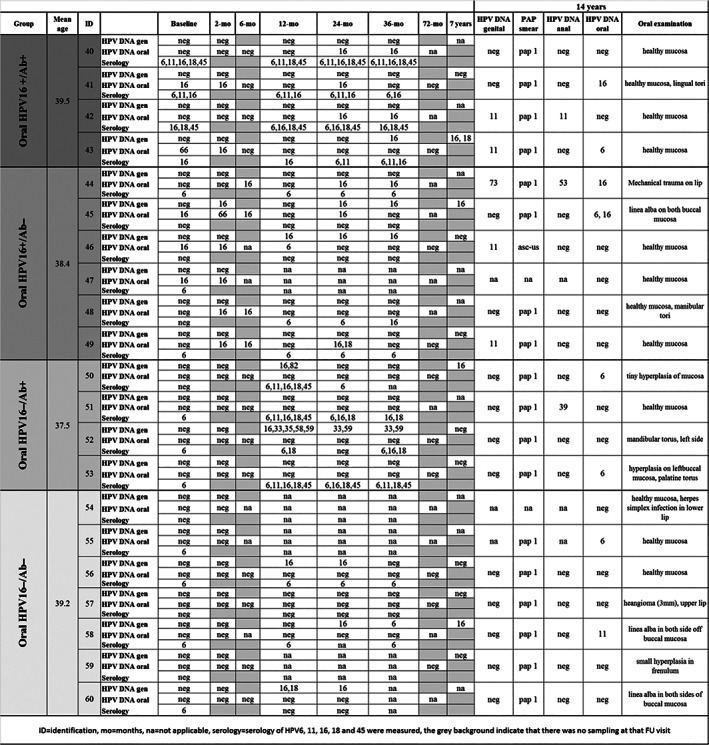
The figure summarizes the women's HPV‐specific genital, oral, and serology data during the 7 years of the follow‐up (FU). At the 14‐year time point of FU, the HPV genital, anal, and oral DNAs were analyzed. Pap smears were also taken. Oral examinations were conducted by a dentist

The women in the present study represent a subgroup of the Finnish Family HPV Study. This study was conducted at the Department of Oral Pathology, Institute of Dentistry, University of Turku, as well as Department of Obstetrics and Gynecology, Turku University Central Hospital (Finland). The study design and its amendments were approved by the Research Ethics Committee of Turku University Hospital (#3/1998 and 45/180/2010). Originally, during the years 1998–2001, 329 pregnant women in their third trimester of pregnancy and all their newborns (*n* = 331; includes two pairs of twins) were enrolled in the study. Enrollment has been described previously (Rintala et al., 2005; Rintala et al., 2006). In addition, 131 male spouses participated in the study (Kero et al., 2012). For the present study, four subgroups of women (*n* = 21) were individually invited based on the known FU data on their oral HPV DNA and HPV serological status as follows: group 1 (*n* = 4), oral HPV16 DNA+/Ab+, that is, women with persisting HPV16 DNA for at least 24 months and also had HPV16 L1‐specific antibodies tested with the multiplex HPV serology analysis; group 2 (*n* = 6), oral HPV16+/Ab−, that is, women with persistent oral HPV16, but HPV16‐seronegative; group 3 (*n* = 4), oral HPV16−/Ab+, that is, constantly HPV16 DNA‐negative but HPV16‐seropositive; and group 4 (*n* = 7), oral HPV16−/Ab−, that is, those being both oral HPV16 DNA‐ and seronegative. Written informed consent was obtained from all women. The mean age of the women at the time of CMI testing was of 38 years 7 months (range: 34–45 years), with no significant differences between the four groups. Their FU data of the women are summarized in Figure 1.

### 2.2 Oral samples

A subgroup of mother–child pairs were recalled 10–14 years after the entry into study for HPV specific CMI studies. At this visit, venous blood samples were taken for CMI in addition to oral brush sampling for HPV testing both from mothers and children. Briefly, the buccal mucosa of the cheeks and vestibular areas were brushed with a Cytobrush® (MedScan, Malmö, Sweden). The Cytobrush was then inserted into 80% ethanol and instantly frozen and stored at −70°C until analyzed. A dentist specialized in oral mucosal diseases (JR) also carried out a comprehensive clinical examination of all women.

### 2.3 Cervical specimen

Gynecological examination was also conducted at the 14‐year FU visit by a gynecologist (KK), including a Pap smear and additional cervical sampling with a Cytobrush® for HPV testing. The cytobrush for HPV testing was placed in a tube with 0.05 M phosphate‐buffered saline (PBS) with 100 μg of gentamycin, immediately frozen at −20°C. Then the Cytobrush was stored at −70°C until tested (Rintala et al., 2005). Three of the 21 women declined the gynecological examination and sampling.

### 2.4 Anal samples

Brush samples (Cytobrush®) were also taken from the anus by the gynecologist. Xylocain gel (AstraZeneca AB, Södertälje, Sweden) was applied to the anal region before sampling to increase the acceptability of the sampling device. The process of the samples was similar to that of the gynecological samples. Three of the 21 women refused the anal sampling.

### 2.5 HPV DNA detection

In addition to the last endpoint sampling, oral brush samples were taken at baseline (before the delivery of the baby) and at 2‐, 6‐, 12‐, 24‐, 36‐, and 72‐months. Cervical samples for HPV testing were collected at the baseline, 6‐, 12‐, 24‐, 36‐, and 72‐months of FU (Rintala et al., 2005; Rintala et al., 2006). The DNA extraction and HPV testing have been detailed in previous reports (Rautava et al., 2012; Rintala et al., 2005). Briefly, HPV DNA was extracted from the scrapings using Miller's high salt method (Miller et al., 1988). HPV testing was performed with nested polymerase chain reaction using My09/My11 and GP05+/GP06+ primers. The multiplex HPV genotyping kit (Multimetrix; Progen Biotechnik GmbH) was used for genotyping. The test identifies 24 low‐risk (LR‐) and high‐risk (HR‐) HPV genotypes: LR‐HPV: 6, 11, 42, 43, 44, and 70; HR‐HPV: 16, 18, 26, 31, 33, 35, 39, 45, 51, 52, 53, 56, 58, 59, 66, 68, 73, and 82 (Paaso et al., 2011; Schmitt et al., 2006).

### 2.6 HPV antibody screening

Blood samples for antibody determination were taken in a clot activator tube at baseline and at 12‐, 24‐, and 36‐months of FU. At first, the samples were centrifuged at 1150*g* for 10 min (Sorval GLC‐2; DuPont instrument). Then the serum was divided into three 1 ml aliquots and stored at −20°C for no longer than 1 week. Until being sent for analysis at the DKFZ, Heidelberg, Germany, the samples were stored at −70°C. Multiplex HPV serology based on glutathione S‐transferase fusion‐protein capture on fluorescent beads (Syrjänen et al., 2009; Waterboer et al., 2005) was used to analyze antibodies to the major capsid protein L1 of HPV types 6, 11, 16, 18, and 45. The median reporter fluorescence intensity (MFI) of at least 100 beads was computed for each bead set in the sample with the cut‐off value separately defined for each HPV probe as 1.5× background MFI + 5 MFI. The cut‐off for HPV seropositivity was MFI 200.

### 2.7 Blood samples for CMI

The isolation of peripheral blood mononuclear cells (PBMCs) was performed as described in Koskimaa et al., 2014 and Paaso et al., 2015). Venous blood samples (74 ml) were collected in sodium‐heparin collection tubes. The isolation was performed by centrifugation for 3 h over a Ficoll‐Paque gradient (GE Healthcare Life Sciences, Uppsala, Sweden). Around ~10 × 10^6^ PBMCs were used for the lymphocyte stimulation test (LST). The leftover cells were cryopreserved in 80% Fetal Bovine Serum (FBS, Biowest, EU quality) and 20% DMSO (Merck, Darmstadt, Germany) at a density of 10 million PBMCs/vial. Autologous serum was used for the cell cultures in the short‐term T cell proliferation assay (Koskimaa et al., 2014; Paaso et al., 2015).

### 2.8 HPV16 peptides and memory response mix

Overlapping 30–35‐mer peptides which covered the entire HPV16 E2, E6, and E7 protein sequences were used in the short‐term LST. This has been previously described (de Jong et al., 2004; Welters et al., 2008). The peptides were synthesized using a solid phase peptide synthesis (SPPS) method with >95% purity (ChinaPeptides Co., Shanghai, China), with a 14 (for 30‐mer) or 15 (for 35‐mer) amino acid (aa) overlapping region. Two pools of E2 peptides (E2.1 and E2.2) comprised 12 or 11 (30‐mer) peptides, respectively. Four pools of E6 (E6.1–E6.4) and two pools of E7 peptides (E7.1 and E7.2) consisted of two 32‐mer or 35‐mer peptides, respectively (Koskimaa et al., 2014). Mass spectrum and high‐performance liquid chromatography (HPLC) was used to test the peptide quality. Memory response mix (MRM) stock solution (50×) was a positive control for the proliferation assay and cytokine production capacity of the PBMCs. It consisted tetanus toxoid, 0.75 fl/ml (Statens Serum Institut, Copenhagen, Denmark), Tuberculin PPD, 5 μg/ml (Statens Serum Institut), and *Candida albicans*, 0.015% (Greer Laboratories, Lenoir, USA).

### 2.9 Proliferative capacity determination of HPV16‐specific T cells by short‐term lymphocyte stimulation test

The protocol for LST is described in more detail in our previous communications (Koskimaa et al., 2014; Paaso et al., 2015). Briefly, the PBMCs were seeded into U‐bottomed 96‐wells microtiter plates (Nunc, Roskilde, Denmark) at the density of 1.5 × 10^5^/well, and for each peptide pool (5 μg/ml at final concentration) eight replicative wells were tested. The culture media was Iscove's Modified Dulbecco's Media, IMDM (Gibco, Life Technologies, Belgium) with 10% autologous serum. PBMCs cultured with MRM were used as a positive control. PBMC cultures with no antigen (medium‐only) served as negative background controls. The supernatants of all eight replicative wells (50 μl/well) were collected 6 days after culturing and pooled for cytokine analysis. ^3^H‐thymidine (PerkinElmer, Turku, Finland) was added at a concentration of 10 μCi/ml (50 μl/well) for the last 18 h of the incubation. Then, the cells were harvested using the FilterMateTM Cell Harvester (PerkinElmer). The filter plates were counted on the 1450 MicroBeta+ counter (PerkinElmer). The average counts plus 3×SD was used for cut‐off value for counts per minute (CPM) values of the eight medium‐only control wells. The calculation of stimulation index (SI) was the average of the tested eight wells divided by the average of the medium‐only control wells. The proliferative response was considered positive if the CPM values of at least six of the eight wells were above the cut‐off value and the SI was ≥3 (de Jong et al., 2002; Welters et al., 2003).

### 2.10 Analysis of cytokine secretion

At day 6, the supernatants were collected from the samples which were LST‐positive. The Cytometric Bead Array (CBA) Human Enhanced Sensitivity Flex Set system (BD Biosciences, Temse, Belgium) was used to determine the levels of IFN‐γ, TNF‐α, IL‐2, IL‐5, IL‐10, and IL‐17A (Koskimaa et al., 2014). As described by the manufacturer, the detection limits for the cytokines were based on standard curves followed with the limit of 274 fg/ml. The limit of a positive antigen‐induced cytokine production was determined as a cytokine concentration exceeding twice the concentration of the medium‐only control was considered as the limit of a positive antigen‐induced cytokine production (Heusinkveld et al., 2011).

### 2.11 Statistical analysis

The IBM SPSS® (IBM, Inc., New York, USA) software package (IBM SPSS Statistics for Windows, version 23.0.0.2) was used for statistical analyses. A one‐way ANOVA was used to calculate the means of secreted cytokine concentrations and proliferative responses of all groups. Multiple comparisons between the groups were controlled with the Bonferroni correction. Differences between the proportions of positive LST responses were calculated with the chi‐square test. All statistical tests were two‐sided. The results were declared significant with a *p*‐value of ≤0.05.

### 2.12 Laboratory environment

T cell assays were performed at the research laboratory of the Department of Oral Pathology at the Institute of Dentistry, Faculty of Medicine, University of Turku, Turku, Finland. The T cell assays were performed according to Standard Operating Procedures (SOPs) with the predefined criteria for positive responses.

## 3 RESULTS

### 3.1 HPV‐specific information during the FU

All HPV‐specific data of the 21 women followed up for 14 years are given in Figure 1, which also includes the results of HPV testing of anal, genital and oral HPV samples collected at the last visit when also the blood sample was taken for CMI analyses. Also, the results of the clinical examination of oral mucosa at the same visit are given in Figure 1. Three of the women with persistent oral HPV16 infection also had HPV16 DNA detectable in their last sample. The majority of the women were HPV‐DNA‐negative for both genital and anal samples. HPV11 was the most common genotype in the genital tract, and there were only three HPV DNA‐positive samples of the anus. Oral HPV DNA was detected in 8/21 women who were positive for HPV6, 11, or 16 (Figure 1).

### 3.2 The HPV‐specific proliferative T cell response is stronger in HPV‐DNA‐negative women

**FIGURE 2:**
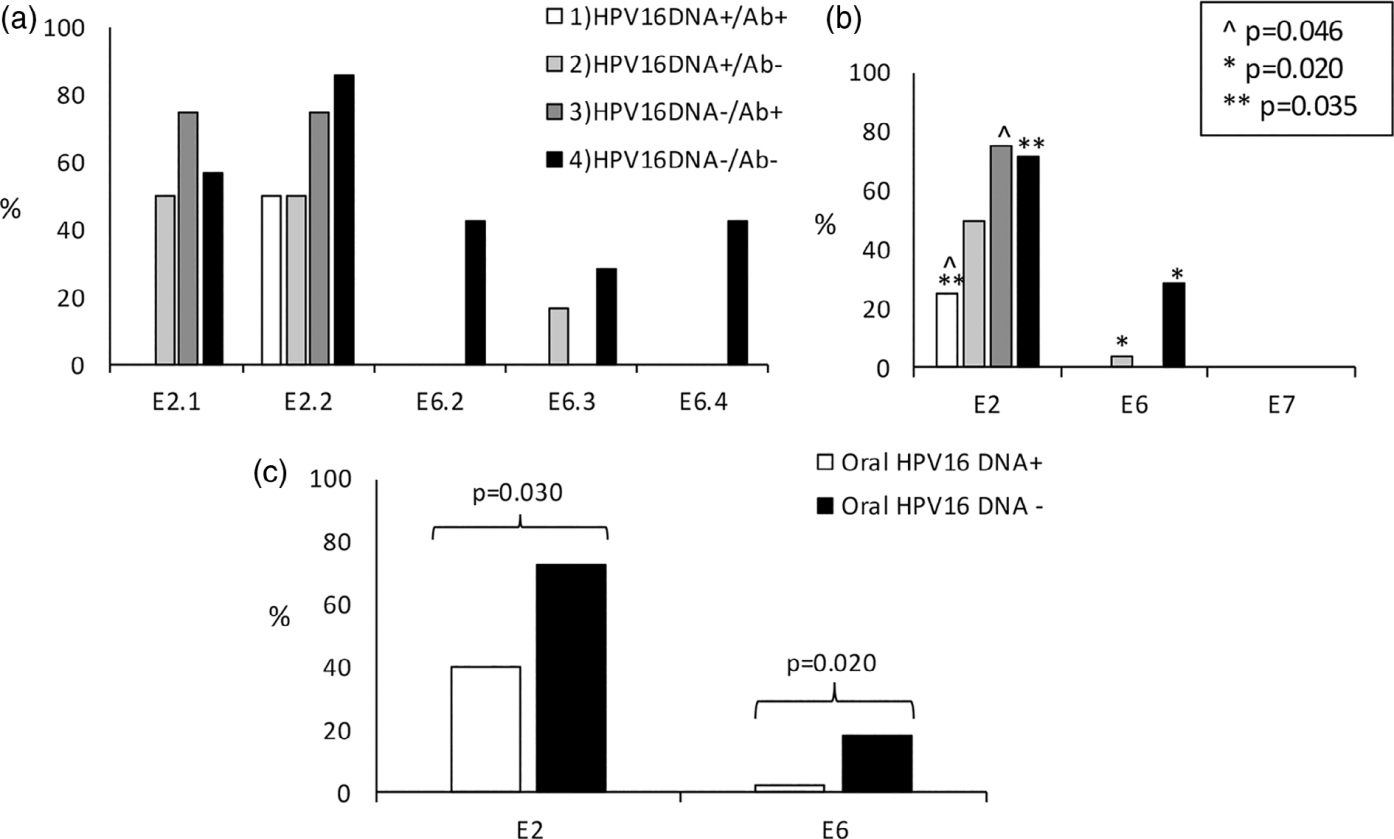
Percentages of positive lymphocyte stimulation test (LST) responses. (a) Positive LST responses as percentages after HPV16 E2.1, E2.2, E6.2, E6.3, and E6.4 peptide pool stimulation in four subgroups. In cell culture, eight replicative wells were used for every peptide pool. Each bar is presenting the positive responsiveness for specified HPV peptide pool as a proportional distribution in different study groups. (b) Positive LST responses as percentages after HPV16 E2‐, E6‐, and E7‐specific stimulation in four study groups. (c) Positive LST responses as percentages using the combined results of E2.1 and E.2 peptide pools similar as with the results of E6.2, E6.3, and E6.4 peptide pools. Subgroups of (1) HPV16 DNA+/Ab+ (*n* = 4) and (2) HPV16 DNA+/Ab− (*n* = 6) were combined to create a group of oral HPV16‐DNA‐positive women. Subgroups of (3) HPV16 DNA−/Ab+ (*n* = 4) and (4) HPV16 DNA−/Ab− (*n* = 7) were combined to create a group of oral HPV16‐DNA‐negative women

Women tested for HPV16 E2‐, E6‐, and E7‐specific peptide pools stratified into the four subgroups: (1) oral HPV16 DNA+/Ab+, (2) oral HPV16+/Ab−, (3) oral HPV16−/Ab+, and (4) oral HPV16−/Ab−. Most of the proliferative responses were detected against the E2.1 and E2.2 peptide pools. Particularly group 4 showed E6‐specific proliferative responses. No E7‐specific proliferation was detected in any of the four subgroups (Figure 2a).

**FIGURE 3:**
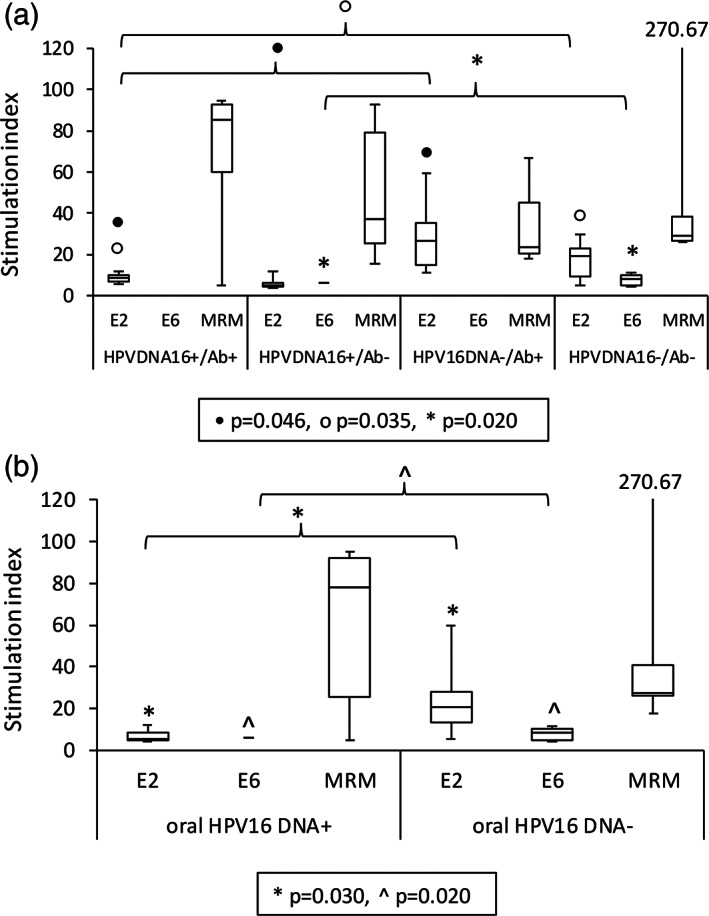
Levels of proliferative T cell responses given as stimulation indexes (SI) against peptide pools of HPV16 E2 and E6 in 21 women. The SI values against the E2.1 and E2.2 peptides were combined similarly as with E6.2, E6.3, and E6.4 peptides. HPV16 E7 peptides did not result on specific lymphocyte stimulation (LST) in any of the subgroups. Values are presented as follows: The box is outlined on the top by the third quartile, the bottom by the first quartile, and in the middle is the median. The minimum and maximum SI values of each subgroup are marked with the whiskers. (a) SI values of four subgroups studied, (b) SI values of oral HPV16 DNA‐positive and ‐negative women

As to the total response to E2 or E6, the strength of the proliferative response (Figure 3) and the percentage of individuals responding to E2 were higher in HPV16 DNA negative groups (groups 3 and 4) than in HPV16 DNA positive group (group 1) (*p* = 0.046 and *p* = 0.035, respectively). The proliferative response (Figure 3) and the percentage of patients responding to the complete E6 were higher in group 4 than in any other group (group 4 vs. 2; *p*‐value = 0.020) (Figure 2b). The women were then grouped together according to their oral HPV16 DNA+/− status (groups 1 and 2; groups 3 and 4). The strength of the proliferative response (Figure 3) and the percentage of women responding to either E2 or E6 were higher among the oral HPV16 DNA− groups; *p* = 0.030 and 0.020, respectively, than among the HPV16 DNA+ women (Figure 2c).

### 3.3 HPV16 reactivity in HPV16 DNA+ women is associated with a dominant IL‐10 and IL‐17A

The cytokine levels of IFN‐γ, TNF‐α, IL‐2, IL‐4, IL‐5, IL‐10, and IL‐17A were analyzed after stimulation with HPV16‐specific E2, E6, and E7 peptide pools. The cytokine responses were determined only in the cultures displaying HPV16‐specific proliferation in the lymphocyte proliferation test. HPV‐specific cytokine responses were observed in all groups. There was no IL‐4 secretion in any group.

**FIGURE 4:**
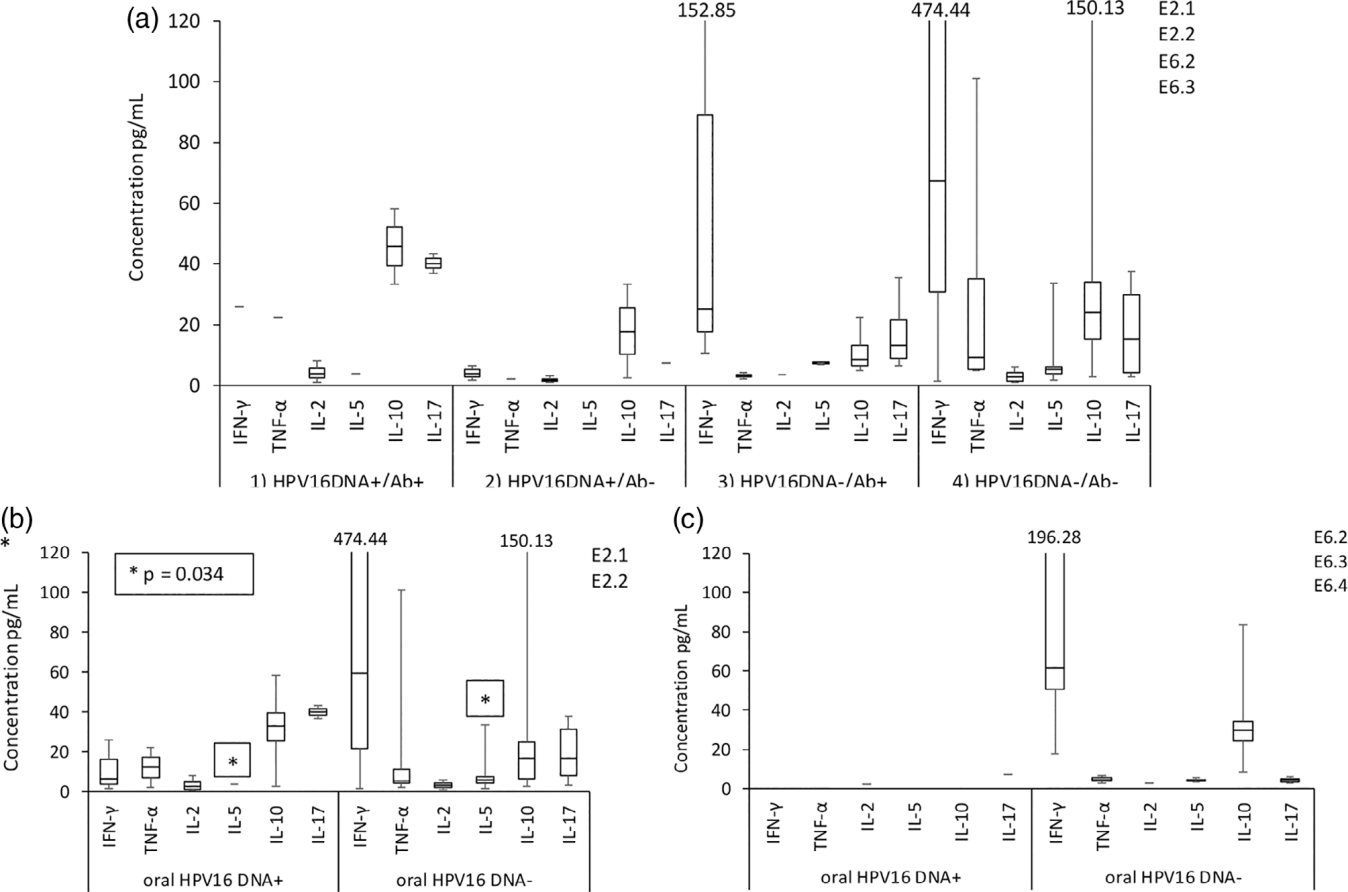
Cytokine concentrations after stimulation with HPV16‐specific peptides displayed in two or four subgroups. (a) The pooled cytokine concentrations after stimulation of HPV16 E2.1, E2.2, E6.2, E6.3, and E6.4 peptides in the four subgroups studied. Each cytokine was measured in eight replicative wells. The number of women in the subgroups was as follows: (1) HPV16 DNA+/Ab+ (*n* = 4), (2) HPV16 DNA+/Ab− (*n* = 6), HPV16 (3) HPV16 DNA−/Ab+ (*n* = 4), and (4) DNA−/Ab− (*n* = 7). The cytokine values are given as pg/ml and the boxes are outlined on the top by the third quartile, the bottom by the first quartile, and in the middle is the median. The minimum and maximum cytokine concentrations of each subgroup are marked with whiskers. (b) The pooled cytokine concentrations after stimulation of E2.1 and E2.2 peptides of oral HPV16‐DNA‐positive (*n* = 10) and ‐negative (*n* = 11) women, (c) the pooled cytokine concentrations after stimulation of E6.2, E6.3, and E6.4 peptides of oral HPV16‐DNA‐positive (*n* = 10) and ‐negative (*n* = 11) women

The total amount of each cytokine produced in all HPV‐specific proliferative cultures of a woman was calculated. The HPV‐specific reactivity of the women in group 4 and group 3 was associated with the highest cytokine levels as compared to the two other groups. IFN‐γ and IL‐10 had the highest levels in group 4 and IFN‐γ and IL‐17A in group 3. IL‐10 was the dominant cytokine produced by the HPV‐specific T cells in groups 1 and 2 (Figure 4a). Subgroup analysis showed that women who were HPV16 DNA‐negative during the FU secreted more IFN‐γ after E2‐peptide stimulation (Figure 4b) or after E6‐peptide stimulation (Figure 4c) than women who were HPV16 DNA‐positive. On the contrary, the HPV16 DNA‐positive women secreted more IL‐10 and IL‐17A (Figure 4a).

### 3.4 Oral HPV16 DNA negative women were characterized by prominent proportional ratios of IFN‐γ and interleukin‐5 secretions

**FIGURE 5:**
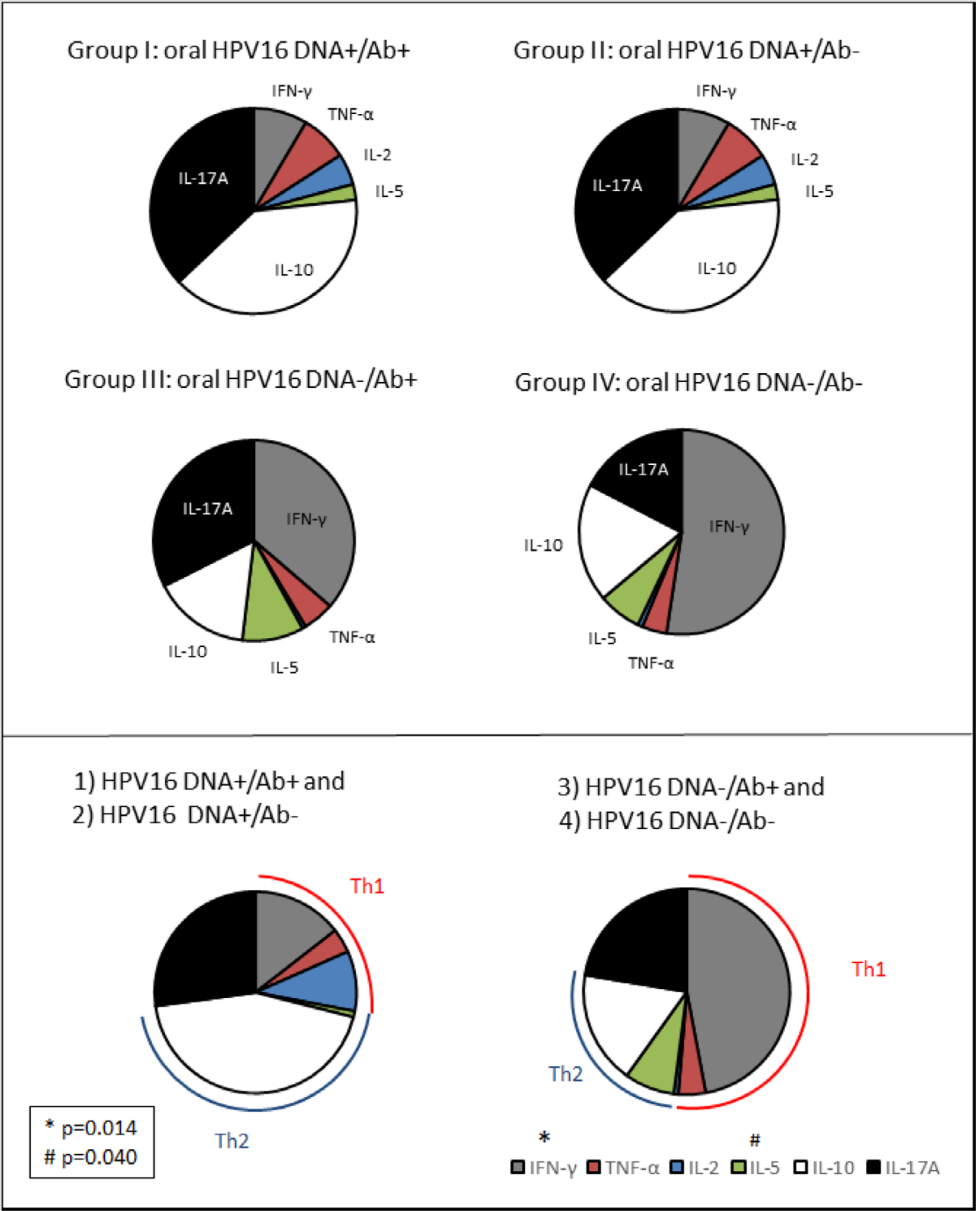
Proportional ratios of the cytokines measured in the lymphocyte stimulation test (LST)‐positive samples. In the upper part of the figure, the women were divided into four subgroups: HPV16 DNA+/Ab+ (*n* = 4), HPV16 DNA+/Ab− (*n* = 6), HPV16 DNA−/Ab+ (*n* = 4) and HPV16 DNA−/Ab− (*n* = 7). In the lower part of the figure, the smaller subgroups were combined to create two groups according to the oral HPV DNA status (oral HPV16‐DNA‐positive (*n* = 10) and ‐negative (*n* = 11) women). In each study subject, single‐cytokines were summed up from the LST‐positive samples, only. Then, the sum value of each cytokine was divided by the total cytokine sum values within the LST+ samples to obtain the cytokine‐specific ratios. Finally, the mean values of the given ratios were calculated for each subgroup

In addition, the proportional ratios of each cytokine were analyzed (Figure 5). From each woman, each cytokine was summed from the LST‐positive (E2 and E6 peptides were pooled together) samples. The mean values were calculated of both the LST+ and LST− samples. After this, the LST+ cytokines were divided by the cytokine values of the LST+ and LST− samples. At the end, for each subgroup the mean values of the given ratios were calculated. In Groups 3 and 4, the most predominant cytokine was IFN‐γ. In contrast, the T cells in groups 1 and 2 mostly secreted more Th2‐ and Th17‐type cytokines like IL‐10 and IL‐17A than Th1‐type cytokines. When the HPV16 DNA+ and HPV16 DNA− women were compared, there were statistically significant differences in the ratios of IFN‐γ (HPV16 DNA+ women: 0.038, HPV16 DNA− women: 0.230, *p* = 0.014) and IL‐5 (HPV16 DNA+ women: 0.001, HPV16 DNA− women: 0.041, *p* = 0.040) (Figure 5).

## 4 DISCUSSION

The number of subjects in this study was small. Despite this fact, we were able to demonstrate differences in HPV16 specific CMI between HPV negative women and the women with persistent oral HPV16 infection. Oral HPV negative women showed strong proliferative E2‐ and E6‐specific responses. These responses were associated with prominent IFN‐γ and IL‐5 secretion.

Oral cavity is the first possible route of HPV infection, especially in children, and could even be the first anatomic site of productive infection resulting in HPV‐specific immunity (Koskimaa et al., 2012; Koskimaa et al., 2014). Our main interest was to find out whether any CMI‐specific indicators could predict the outcome of oral HPV16 infections. Thus, based on their oral HPV16 DNA and HPV16 serology status during the FU the women were classified into four subgroups. We hypothesized that the HPV‐specific immune system is compromised in women with persistent HPV16 infection and lack of protective HPV‐specific antibodies (group 2) during the FU. Those women might be at increased risk for HPV induced oral mucosal lesions—and even cancer—development. As expected, E2‐specific proliferation was most common among the HPV16 DNA‐negative women (groups 3 and 4). In unison, E6‐specific responsiveness was predominantly found in group 4. It was previously found that reactivity against E2 and E6 is typical for HPV‐negative persons. This explains why circulating CD4+ and CD8+ T cells specific for HPV16 E2 and E6 antigens are frequently detected in healthy subjects (i.e., devoid of genital HPV) who have successfully cleared the virus (de Jong et al., 2002; Welters et al., 2003). The fact that none of the women had clinical oral lesions could be explained with the lack of E7‐specific proliferation. Among HPV‐positive oropharyngeal or CC patients, E2‐specific proliferation is less prevalent, but E6‐ and E7‐specific T cell proliferation is detected in many cases (de Jong et al., 2004; Piersma et al., 2008). This difference in immune reactivity suggests the best protective CMI against progressive oral HPV16 infection for the HPV16 DNA‐ and antibody‐negative women (group 4). While oral HPV16 DNA has never been detected in those women during the whole FU, it does not rule out the possibility of a subclinical HPV16 infection at some point of their life. The presence of HPV antibodies in sera was the difference between the two HPV16 DNA‐negative groups 3 and 4 and the absence of E6‐reactivity in the former. The presence of antibodies indicates a previous exposure to HPV16, which was not resolved in time to prevent the development of antibodies (Sasagawa et al., 1998). Potentially, the lack of E6 reactivity, which is usually found in (HPV antibody‐negative) healthy individuals, is related to this. For genital infections, a role for E2‐ and E6‐specific T cells in resolving the early stages of HPV infection has also been suggested (Dillon et al., 2007; Farhat et al., 2009; Paaso et al., 2015; Welters et al., 2003; Woo et al., 2010).

In addition to Th1/Th2 cytokines, also IL‐17 cytokines (mainly IL‐17A) produced by Th17 T cells play important role in host immune responses during the elimination of pathogens. IL‐17 might also stimulate the production of IFN‐γ and TNF‐α by T cells and natural killer (NK) cells which amplifies the inflammation. Therefore the persistent production of IL‐17 is a risk factor for chronic inflammation and elevated levels of IL‐17 have been found in CC patients (Chen et al., 2013; Zhang et al., 2011). We measured lower concentrations of IL‐17A among women with oral persistent HPV16 infection than among the HPV‐negative control women and the overall concentrations were low. The most probable reason for divergent results is the small sample amount and other possible explanation might be the stage of HPV infections. In other studies, the divergences have been able to found when comparing cancer patients, women with different stages of CIN and healthy controls (Xue et al., 2018). All women in our study are basically healthy without any clinically detectable lesions in mouth or squamous intraepithelial (SIL) in pap smears.

The most protective cytokine combination seem to be with oral HPV16 DNA− and Ab− women (group 4). The mixed Th1/Th2‐type cytokine secretion is typical to healthy women compared to CC patients (de Jong et al., 2004; Welters et al., 2003; Welters et al., 2006). In line with these previous results we could show evidence that the proportional ratios of IFN‐γ and IL‐5 cytokine secretions were significantly higher also in the oral HPV16 DNA‐negative women than in oral HPV DNA persistors (de Jong et al., 2004). Some previous evidence suggests that IFN‐γ is one possible prognostic marker for the HR‐HPV clearance (Scott et al., 1999; Welters et al., 2003). Our previous study also showed that women who tested HPV negative in genital tract or who could clear their HPV infection had higher levels of IFN‐γ after HPV16 peptide stimulation than women with HPV induced cervical intraepithelial lesions (CIN) (Paaso et al., 2015). In line with our results, Ondondo and coworkers recently reported that men with HPV clearance had significantly higher IFN‐γ levels than those with persistent HPV infection (Ondondo et al., 2019). They concluded that Th1 cell‐mediated cytokine response was associated with natural HPV clearance in men (Ondondo et al., 2019). Similarly, they found that HPV IL‐2 levels were higher in HPV clearance group then in noninfected men which was however, not evident in our study. Contradictory to us, they used L1 peptides for peripheral blood lymphocyte stimulation which signifies better for productive HPV infection than stimulation with E2, E6, or E7 peptides.

Increased IL‐10 levels have been identified in CIN lesions. T cell activation and Th1 cell differentiation can be inhibited by IL‐10 (Alcocer‐Gonzalez et al., 2006; de Jong et al., 2004; Lin et al., 2019; Prata et al., 2015). During the virus infection the role of IL‐10 is crucial. Many cell types are able to secrete IL‐10 and the major source of IL‐10 is varying depending on the stage of infection (acute or chronic). For example, through co‐operation with cytokines of Th1, the IL‐10 is able to regulate Th2 cytokines like the overproduction of IL‐4 and IL‐5 (Couper et al., 2008). The highest concentration of IL‐10 after HPV16 peptide exposure was secreted by the peripheral blood mononuclear cells derived from women with persistent oral HPV16 infection and HPV16 seropositivity (group 1). Despite our limited amounts of results, we assume that oral HPV 16 infections could affect the host's CMI similarly as described by the earlier CMI studies focused only on HPV infections in the cervix, so far.

To conclude, our results indicate a mixed Th1/Th2/Th17 cytokine profile while in oral HPV16 persistors the proliferative E2 and E6 responses were partly impaired and lacked the IFN‐γ and interleukin‐5 secretion. Also the proportional ratios of cytokines measured from LST‐positive samples indicate a slight shift toward Th2/Th17 profile, similarly as found earlier in patients with CC or severe CIN (de Jong et al., 2004; Lin et al., 2019). However, here the differences were less prominent as we studied here women with chronic oral HPV infection but clinically healthy during a long FU (de Jong et al., 2004).

In the future, there is an urgent need for additional studies on patients suffering from persistent HPV infections with SIL and carcinomas in head and neck region especially in oral, oropharyngeal, and sinonasal tracts which might be the first sites of HPV infection in early life. Furthermore, these areas are in or adjacent to lymphoid tissues facilitating an early recognition of infectious agents HPV included.

## AUTHOR CONTRIBUTIONS

Anna Paaso, Stina Syrjänen, Kari Syrjänen. Data collection: Anna Paaso, Hanna‐Mari Koskimaa, Katja Kero, Jaana Rautava concepted and designed the work. Anna Paaso, Hanna‐Mari Koskimaa, Marij J. P. Welters, Katja Kero, Jaana Rautava, Kari Syrjänen, Sjoerd H. van der Burg, Stina Syrjänen analysed and interpretated the data. Article was drafted by Anna Paaso, Hanna‐Mari Koskimaa, Katja Kero, Jaana Rautava. The article was critically revised by Marij J. P. Welters, Kari Syrjänen, Sjoerd H. van der Burg, Stina Syrjänen. The final version to be published was approved by Anna Paaso, Hanna‐Mari Koskimaa, Marij J. P. Welters, Katja Kero, Jaana Rautava, Kari Syrjänen, Sjoerd H. van der Burg, Stina Syrjänen.

## CONFLICT OF INTEREST

There is no conflict of interest.

## Data Availability

The data that support the findings of this study are available from the corresponding author upon reasonable request.
